# 

**Published:** 1888-03-31

**Authors:** 


### QUESTIONS AND ANSWERS.

### SILVER EAR DRUMS.

Will any reader of The Hospital kindly tell Sister Grace where Silver Ear Drums may be had, and if any particular maker can be recommended?

### SPECIAL NOTICE TO NEWSAGENTS, ADVERTISERS, AND THE TRADE.

### CHANGE OF OFFICE AND PUBLISHER.

Notice is hereby given that the Office of The Hospital will henceforth be at 38, Old Jewry (Cheapside end), E.C., to which address all communications should be sent. Mr. A. Doig has been appointed Advertising Agent, and Mr. J. H. S. Hanning, Manager. All Cheques and Post Office Orders should be made payable to J. H. S. Hanning, 38, Old Jewry, E.C. crossed Lloyds, Barnetts, and Bosanquets Bank (Limited).

### TO SUPERINTENDENTS, SECRETARIES, AND MATRONS.

The Editor will be glad to have a copy of the Regulations, and also of every Report issued from the Press of Asylums, Hospitals, Convalescent and Nursing Institutions, as well as those of other Charities, so soon as they are published, and an early copy in duplicate of all appeals for funds. Notices of Annual and other Meetings, Festival Dinners, etc., will be welcomed. All such communications should be addressed to *The Editor, The Lodge, Porchester Square, London, W.* Any superintendent, secretary, matron, or other chief official who will regularly send such documents and information will be registered to receive free of cost a cover for binding The Hospital in half-yearly volumes on sending name and address to the publisher.

### PERMANENT ENLARGEMENT OF "THE HOSPITAL."—NEW ARRANGEMENTS FOR VOLUME IV.

It has been determined to publish a special "Nursing Supplement" with every number of The Hospital, commencing with our issue of April 7th. This has been decided with the view of giving all who are engaged in nursing opportunities of inter-communication, and it is hoped that lady superintendents, matrons, sisters, and nurses will co-operate with the Editor in making this special supplement instructive and interesting. Contributions on nursing topics, including intimations of vacancies, changes in the nursing staff, appointments, examinations and examination papers, the award of prizes and medals, abstracts of cases taken by nurses (but confined entirely to the nursing), and all similar matters, will be welcomed. Short articles upon subjects affecting nurses of all degrees are invited, and, if accepted, will be paid for. Offers to take charge of any section of the special Supplement, together with the communications intended for it, should be addressed to the Editor, The Lodge, Porchester Square, London, W.

### NEW SERIAL STORY.

A new serial story, by Leslie Keith, entitled "An Ishmaelite," will be commenced in The Hospital of April 7th. The author, who has won wide popularity by his writings, has surpassed himself in this new story, which will be found to be of exceptional interest and power. Those who wish to become subscribers to The Hospital for the next volume should give their orders at once, to avoid disappointment and delay, owing to the great and increasing demand which exists for the paper at the present time.

### THE NATIONAL PENSION FUND.

The prospectus of this fund, showing the premiums payable for pensions only, and for pensions with sick allowance, have now been finally approved, and will be ready to be issued after Easter. Next week's Hospital will contain a summary of the prospectus and tables, with a descriptive article based on the actuary's report, to enable our readers to thoroughly master the details of the scheme. It will be found that an attempt has been made to meet all requirements in this prospectus, and that the Council promise that special cases, not actually provided for, will be carefully considered on written application being made to the Secretary of the Fund, at 38, Old Jewry, E.C.

### AMUSEMENTS WITH PROFIT FOR ALL AGES.

**Rules.**—All answers must be addressed to the PRIZE EDITOR, THE HOSPITAL, 38, Old Jewry, Cheapside, London, E.C., not later than he *first* post on Thursday next. The full name and address must in all cases be given, but a *nom de plume* may be added for publication. Only one side of the paper must be written on.

### FOR MEN AND WOMEN.

**Rule.**—Competitors can enter for *all* quarterly competitions, but no competitor may take more than *two quarterly prizes* during the year.

### Acrostic Competition.

Second quarter commenced February 25th, 1888; ends May 19th, 1888.

A PRIZE of ONE GUINEA will be given to the competitor who gains the highest number of marks for best original acrostics during the ensuing quarter. All acrostics sent in for this competition will be credited according to merit, twelve marks being the highest score given for one acrostic.

A PRIZE of HALF-A-GUINEA to be given to the competitor who gains the highest score for solution of acrostics set. *One mark* only will be given to the competitors who solve all the lights of each acrostic.

Exception will be made in the case of *quotation acrostics*, when *two marks* will be given, *one* for the correct solution of all *lights*, and *one* mark for the *source* of the quotations.

This week credit will be given for

**No. 3 Double Original Acrostic.**

RESULTS OF NO. 2 ORIGINAL DOUBLE ACROSTIC.

Patience (10), Brownie (10), K. M. (10), Padre (9), Mater (9), A. M. E. (9), Sister (8).

### (A) THE WORD COMPETITION.

Second Quarterly Competition commenced February 4th, 1888; ends April 28th, 1888.

Three prizes of one guinea, 15*s*. and 10*s*. 6*d*. will be given each quarter to the three competitors who obtain the highest number of marks; (1) by making the largest number of words derived from the word or phrase set for anatomical dissection; or (2) for the best answers to (B); or (3) by (1) and (2) combined.

We shall give the newspapers and periodicals for dissection during this quarter, that for this, the *ninth* week of the quarter, being

"CORNHILL."

OBSERVE.—Proper names, abbreviations, foreign words, words of less than four letters, and repetitions are barred; plurals, and past and present participles, of verbs are allowed. Nuttall's *Standard* dictionary only to be used.

N.B.—All words sent in must be arranged *alphabetically*, with correct total affixed.

**Results of Seventh Week.**

*Contemporary.*—Ellice (446), Gretta (445), E. E. (442), Patience (431), Sym (422), Nelle (420), Lauriston (420), Lancashire Witch (418), Lodge (412), Butterfly (405), Lightowlers (402), Brisbane (402), Dollie (380), Dunce (325).

### (B) THE LITERARY COMPETITION.

Some people hate puzzles of all kinds, including word competitions, but they are fond of correspondence, and many sisters and mothers are continually receiving and sending letters to and from members of the family who are abroad in India or the Colonies. The constant coming and going between England and other portions of the British Empire make it a matter of interest and importance to know something about the life, the climate, the amusements, the adventures, the clothing, and the surroundings of those who seek a livelihood beyond the seas. We shall therefore try to find space for the publication of selected letters from abroad, which may be sent by any of our readers who have friends and relations in foreign lands. We shall give marks for these letters as well as for the word competitions which will count equally for these prizes, so that anyone may enter for both or either, the prizes to be given to those who get the highest number of marks.

**Letters from Across the Sea.**

*L'Americaine* sends the following extract from a letter from N. S. Wales:—

I am still trying to settle down after our delightful "tramp abroad." C—— won't even *try*, and it certainly is hard to return to such a dull place as this, especially as we came into the middle of the sixth and worst drought. Everything is burnt up, and the whole community suffering from the effects of this continued want of rain. Now things have changed and we are having a grand season, but it will be long before the colony quite recovers. We heard from Y—— a short time ago, and he gave us a sad account of the losses "up country," occasioned by the drought. He had had rather an exciting adventure himself, from the effects of which he was still suffering when he wrote. During his temporary absence from the station, a poor fellow was lost in the bush. Y——, with his usual pluck and kindheartedness, at once set off alone in search of the missing man. He says: "When I came back I found that a man had been away for three days, and no one had been in search of him. So I started immediately. After taking a circle round where he was last seen, I cut his horse tracks and followed them up for two days. At last I found him quite out of his mind. My stock of water, contained in two small bags, was nearly exhausted, for in the hurry of getting off to look for him, I had not realised that I might be absent for some days, and I was frightfully hard up for a drink myself, having been able to take only just sufficient water to keep life in me. He was wild with thirst when I found him, and I gave him all that I could possibly spare. It was a terrible time, and inured to hardship as I am, I believe it will be a long time before I get thoroughly right again. However, I saved the man's life, though I am sadly afraid he will not recover his reason or speech for some time, if ever he does." We fully intend making another trip to the old country whenever we are able to do so, and C—— has been making rash statements as to our *immediate* return to England, ever since we came back, and next time we are to stay two years.

WRITTEN AT SEA.

GRETTA sends the following letter, written on board a passenger s.s. running between Hong Kong and Calcutta:—

Now I am at sea again, I am going to write you all about my ship. She is a beautiful ship, and very comfortable. I will commence the description from forward. On the forecastle are two handsome lighthouses, and below on the forepart of the main deck is one large house: one side has all the officers' rooms, and the other side the engineers' rooms and mess-rooms. My room is particularly nice: mahogany bedstead, muslin curtains, drawers, washstand, cupboard, bookshelf, large looking-glass, water-bottle rack, nice horse-hair couch, lamp, door-screen, ornamental glass window; these, with my clock desk and thermometer, represent my furnished apartment on board. I have a Chinese servant, who has nothing else to do but look after me and keep my room in apple-pie order. Well, after passing our house you come to the sheep pens and pig-stye in front of the lower bridge; then there are two little streets under the bridge on each side, and here on either side are bath-rooms, sculleries, bakeries and numerous other rooms. In the centre of the street is the saloon galley or kitchen, the sailors' galley, the fireman's and the deck or third class passengers' galley, the quartermaster's rooms, cooks', assistants', etc., and a few state rooms. Well, above on the lower bridge is the steam steering house and the chart-room, with a nice sofa and bed in. The captain and myself are the two persons who use this room for the navigation of the ship. In the wheel-house are two dozen rifles and swords, revolvers, signals, &c. We have eight splendid boats, four on either side; four of them are lifeboats. Abaft the bridge on the main deck comes the engine-room with splendid engines, then, right aft to the stern of the ship, comes a very nice saloon on deck which you can enter from both sides and also right aft. There are a nice lot of bath-rooms for the passengers, and a good saloon with punkahs and mirrors, sideboard, etc., and a splendid piano and plenty of nice books to read. The deck over the saloon is all laid out with easy chairs, little tables, &c., all covered with double awnings. The upper bridge is the last part up there. I keep watch night and day. There is plenty of room for a nice walk, telegraph and speaking tubes to the engine room, and a wheel for steering the ship. There are three different wheels, in case one breaks down. In hot weather and when it is fine, I have tiffin and dinner up there. Calcutta is one hundred miles up the river; it is a very large city with fine public buildings. The Supreme Court is nearly as large as our New Law Courts, and not unlike them, and the General Post Office nearly as large as St. Martin's-le-Grand. I will not give you a long description of Shanghai; it is undoubtedly the finest place in the East, a sort of West-end on the river bank, electric lights, splendid roads and nice drives, and by all accounts nice society. It is a treaty port, and divided into three concessions; first, the American; the centre, English; and the end, French. The English is fine, at the commencement are the gardens down to the river, small but exceedingly nice. The band plays most of the year, and you can see as healthy European children there as in any part of the world. More of Shanghai, Swatow, etc., in my next.

### THE CHILDREN'S CORNER.

PRIZES.

FOR MOST CORRECT ANSWERS TO PUZZLES.

This Commenced October 1st, 1887.

One Pound a Month.

A PRIZE OF THREE GUINEAS

to the Competitor who shall have sent in the largest number of correct or best answers during the twelve months.

**Puzzles—Fifth Week.**

No. 17. HISTORICAL CHARADE.—My *first* is a light-covered waggon. My *second* transposed a term for boys. My whole a people who conquered Africa, and tell me under whom?

No. 18. ENIGMA.

Take me entire, my valuable juice
In medicine is found to be of great use;
Divide me, and this such a change does create,
That now I am water in its double state.

No. 19.—Write in ordinary characters the word expressed by 54 E.

No. 20. GEOGRAPHICAL ACROSTIC.—My primals give the name of an island in Europe, my finals something for which is noted. 1. A body of floating ice. 2. A Danish king of England. 3. An English town celebrated for its cathedral. 4. Followers of Wickliffe. 5. An English Queen. 6. A city of Belgium. 7. A town in the Isle of Man

**Results of Third Week Puzzles.**

No. 9. CHARADE. Inn, or moon.

No. 10. DOUBLE ACROSTIC.

W ic K
O u I
O nio N
D arlin G
P uf F
E nged I
C rœsu S
K navis H
E xaggerat E
R owe R

No. 11. CONUNDRUM. A cat has *claw* at the end of its *paws*, a comma its *pause* at the end of its *clause*.

No. 12. TRANSPOSITION. Palm. Lamp.

*All right*—Scrooge, Jumps, Mount Edgecumbe *Three right*—Plummy Fluff, A. C. K., Bagshot, Bookworm, Gem, Squeaker, Excelsior. *Two right*—Spider, Alsie. *One right*—Babette.

**Conditions.**

I. Every paper sent in must be clearly written, and one side only must be used. All competitors must mark at the head of their papers the number of their competition.

II. Each paper must be signed by the author with his or her real name and address. A *nom de plume* may be added, if the writer does not desire to be referred to by us by his real name. In the case of all prize-winners, however, the real name and address will be published.

III. All communications relating to prize competitions should be addressed to "The Prize Editor, THE HOSPITAL, 38, Old Jewry, London, E.C." Competitors are requested not to include in letters, etc., to the Prize Editor communications on matters of other business. All letters must be fully prepaid.

IV. The Prize Editor of "THE HOSPITAL" is the sole judge of the competitions, and his decision is in all cases final and without appeal.

V. The Prize Editor reserves the right to publish any paper sent in, whether it receives a prize or not. He does not hold himself responsible for any MSS. sent, nor can he undertake to return rejected competitions.

VI.—All children resident at school are requested to forward their *home* address in addition to the school one, when entering for the "Children's Corner" Competitions.

**Notice to all Correspondents.**—N.B.—All letters referring to this page, which do not arrive at 38, Old Jewry, Cheapside, London, E.C., by the *first post on Thursdays*, and are not addressed PRIZE EDITOR, will in future be disqualified and disregarded.

No one will be permitted to win the Monthly Prizes twice in any one year, the Annual prizes being offered especially to prevent this.

